# Investigation on the Correlation between Mechanical Strength, Grain Size, and Density of Fly Ash Microspheres in the Context of Refining Process

**DOI:** 10.3390/ma17143459

**Published:** 2024-07-12

**Authors:** Tomasz Radko, Agata Wajda, Tomasz Iluk, Jan Najser

**Affiliations:** 1 Institute of Energy and Fuel Processing Technology, Zamkowa 1, 41-803 Zabrze, Poland; tradko@itpe.pl (T.R.); tiluk@itpe.pl (T.I.); 2 Institute of Clean Technologies for Extraction and Utilization of Energy Resources, Technical University of Ostrava, 17. listopadu 2172/15, 708 00 Ostrava, Czech Republic; jan.najser@vsb.cz

**Keywords:** microsphere, mineral resources, fly ash, cenospheres, mineral waste recovery

## Abstract

Fly ash microspheres, also called cenospheres, have many valuable properties that allow them to be widely used. Some of its most important properties are its mechanical and thermal strength as well as its chemical stability. These features constitute an important commercial parameter. Refining processes aim to select the highest quality product from raw materials that meets the expectations of recipients. Generally, preparing a final product involves selecting the appropriate sequence and parameters of the grain separation process. However, the key to the optimal selection of these parameters is knowledge of the specificity of the processed raw material. Microspheres are materials that are created spontaneously, uncontrolled, and without the possibility of intentionally influencing their properties. Therefore, due to the potential directions of microsphere use, it is justified to study the relationship between density, grain size, and mechanical strength. Understanding these relationships in microspheres from various sources is particularly important at the stage of planning refining processes. This paper presents the results of research on microspheres from two different sources. The tested raw materials (microspheres) are subjected to densiometric and grain analysis. Also, mechanical strength was determined for the separated density fractions and grain classes. The test results did not show significant correlations between the tested features of the microspheres. In the case of both raw materials, the highest density was observed in the smallest grain classes, and the highest mechanical strength was determined for microspheres with grain sizes in the range of 75–100 µm. For this grain size range, the value of mechanical strength is 26 for raw Material 1 and 38 for raw Material 2. The shares of this grain fraction in the microsphere stream are 11.2% and 16%, respectively. An important difference that may significantly affect the efficiency of the refining process is the method of distribution of the primary falling parts, which affects the mechanical strength of the tested raw materials.

## 1. Introduction

Microspheres, also called cenospheres, constitute a specific fraction of fly ash generated in thermal power plants. They are a by-product of a process and are considered as post-process waste with a high recovery potential. These are aluminosilicate grains with low apparent density, which, due to their numerous properties, constitute a valuable raw material [1,2,3]. Microspheres are generated during the combustion of coal in pulverized coal boilers, and the mechanism of their formation is complex and not fully understood. Attempts to explain this phenomenon have been made by, among others, Fenelonov et al. [4] and Danilin et al. [5]. Aluminosilicate grains are spherical in shape and have a gray, light gray, or white color. Microspheres have two distinct parts: a gaseous part filling the interior of the grain and a solid part constituting its outer shell. The interior is mostly filled with CO_2_ and N_2_. The gaseous phase also includes CO, O_2_, and H_2_O, but in significantly smaller amounts. The walls of microspheres have an amorphous structure with slight crystalline inclusions. The outer shell is mainly composed of SiO_2_ and Al_2_O_3_. Other oxides, such as Fe_2_O_3_, CaO, and MgO, are present in much smaller proportions [6,7,8].

The content of microspheres in fly ash depends on several factors. The most important ones are the properties of burned coal and the applied combustion technology. In practice, the most efficient process, in terms of obtaining quantities of aluminosilicate grains, is the energetic combustion of coal in a pulverized coal furnace, where sufficiently high temperatures are present [9,10,11]. In such cases, the proportion of microspheres in the stream of fly ash averages around 1% [4,11].

These are the following methods of obtaining microspheres from combustion residues: wet, dry, and combined. On an industrial scale, the wet method is used due to ease the process implementation, based on the phenomenon of sedimentation under the influence of gravitational forces. First, a layer of aluminosilicate grains is collected on the surface of settling basins, where ash–slag mixtures are discharged. Due to the low density of the microspheres, their separation from other particles occurs spontaneously. Then, the top layer is collected [12,13,14,15].

The most important properties of microspheres include high resistance to chemical factors, as well as high and low temperatures. The microspheres have significant compressive strength and crushing resistance. They have very low wall porosity, which makes them characterized by low water absorption. Thanks to their low thermal conductivity coefficient, they have excellent thermal insulation properties. An increase in temperature causes only small changes in the value of this parameter. Hence, microspheres can be used in a wide range of temperatures [12,16,17,18]. 

Microspheres have many applications. They are widely used in the building materials industry as all kinds of fillers that improve the performance of products. Most often, the purpose of their use is to increase the thermal insulation and fire resistance of products, for example, insulating boards, lightweight concretes, or cement composites [19,20,21,22]. Apart from construction, aluminosilicate grains are used, among others, in the oil industry, mining, energy, automotive, ceramics, and plastics industries. Microspheres can be used to produce brake pads, drilling fluids, and anti-corrosion coatings [17,23,24,25]. It is worth noting that the use of aluminosilicate grains is consistent with the goals of a circular economy. The use of waste materials as substitutes for natural raw materials is highly recommended [26,27].

The main quality parameters of the products are the true density and mechanical strength of the microspheres [28,29,30]. These two parameters determine the commercial value of the products. As microspheres are a product spontaneously formed in pulverized coal boilers fired with coal, it is not possible to influence their formation. At this stage, the type of coal burned, the combustion technology, and the desulfurization technology are of key importance [11,25]. To some extent, it is possible to control their quality as part of the microsphere refining process. The aim of this process is to prepare microspheres obtained from power plants or combined heat and power plants for sale, taking into account the specific needs of a given industry in relation to this product. Among other things, grains of specific sizes, densities, and strengths are released. The variability and instability of the properties resulting from the spontaneous formation of microspheres cause problems with obtaining appropriate parameters for the refined products, i.e., mechanical strength and true density [31,32]. Determining the density and mechanical strength in selected grain size classes is important, among other things, for designing methods of microsphere refinement. Moreover, the separation of appropriate grain size classes should ensure the production of grains with the desired mechanical strength, which in turn allows for achieving higher profits.

This study attempted to establish correlations between selected properties of microspheres, primarily grain size and true density, and their mechanical strength. The subjects of the research are aluminosilicate grains obtained from two sources differing mainly in the type of coal burned. The scope of the conducted research included determining selected properties of the tested microspheres, including the major oxide composition, separation of density fractions and grain classes, and determination of their mechanical strength. Based on this, a comparison of the characteristics of the investigated materials is made. It is also important to know the properties of microsphere raw material in terms of its refining process. By examining the properties of microspheres from different sources, it is possible to forecast certain general tendencies, which will then serve as a basis for designing the enhancement system. For instance, establishing mechanical strength as a key parameter, the raw material preparation system should be adjusted to obtain the highest quantity of high-strength product. This might concern a specific grain size class or density fraction. The collected data will therefore be used to determine certain trends and, based on those, recommendations for technological processes of raw material enhancement aimed at obtaining a product with desired characteristics.

## 2. Materials and Methods

### 2.1. Characteristics of the Tested Microspheres

Two microsphere raw materials are selected for testing and are characterized as follows:

- Material 1—microspheres from Poland, from the combustion process of bituminous coal in a pulverized boiler, with a wet separation process—the sink–float method,
- Material 2—microspheres from Kazakhstan, from the combustion of coal bituminous in a pulverized boiler, with a wet separation process—the sink–float method.

**Figure 1:**
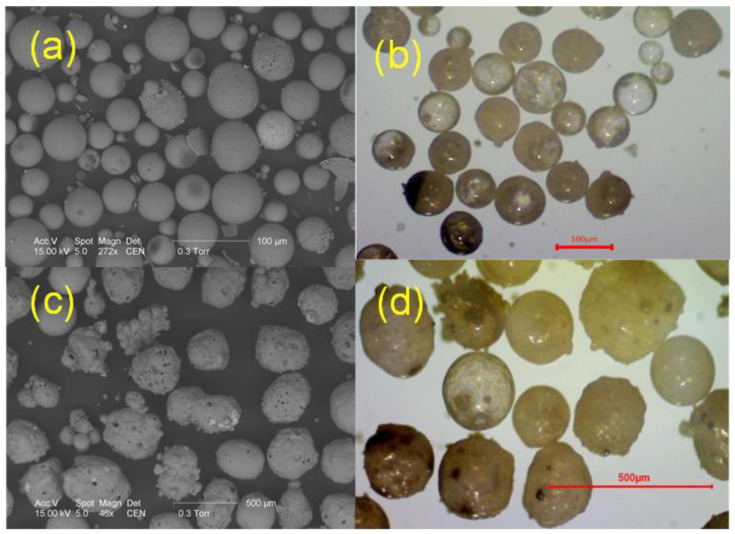
Scanning electron microscope (SEM) and optical microscope (OM) images of microspeheres form tested raw materials: (**a**) SEM image of grain sizes below 100 µm; (**b**) OM image of grain sizes below 100 µm; (**c**) SEM image of grain sizes above 315 µm; (**d**) SEM image of grain sizes above 315 µm.

Figure 1 shows the example of SEM (scanning electron microscope) and optical microscope (OM) images of microspheres from tested raw material. 

The images in Figure 1 show a general view of microspheres with grain sizes below 100 µm, (a) and (b), and above 315 µm, (c) and (d). Microspheres are small spherical particles with different morphology. The smaller particles are mostly transparent, while the larger particles are rather opaque. On the surface of larger grains in Figure 1c,d, a porous structure is clearly visible. It can also be seen that microsphere grains can take on different hues.

### 2.2. Methodology for Investigating Selected Properties of the Microspheres

As part of the research, density fractions and grain classes were separated from samples of selected raw materials mentioned in Section 2.1, analysis of selected physico-chemical properties and determination of their mechanical strength were performed. 

First of all, selected samples of raw materials were dried. It was established that microsphere samples weighing approximately 25 kg need to be dried to constant weight at a temperature of 105 °C in a thermal chamber. At the stage of developing the methodology for testing microspheres, the information that drying microspheres at temperatures above 100 °C may increase the content of falling particles was verified. Comparative tests carried out on drying microsphere samples at temperatures of 80 °C, 90 °C, and 105 °C did not show an increase in the number of falling parts, which would indicate a decrease in mechanical strength (the procedure for determining mechanical strength is described in the next section). 

The following research procedures were conducted as part of laboratory investigations: separation of density fractions, separation of grain size classes, determination of true density, determination of oxide composition using XRF method, and determination of mechanical strength.

The research was conducted based on the following procedures:

(1) Separation of density fractions

**Figure 2:**
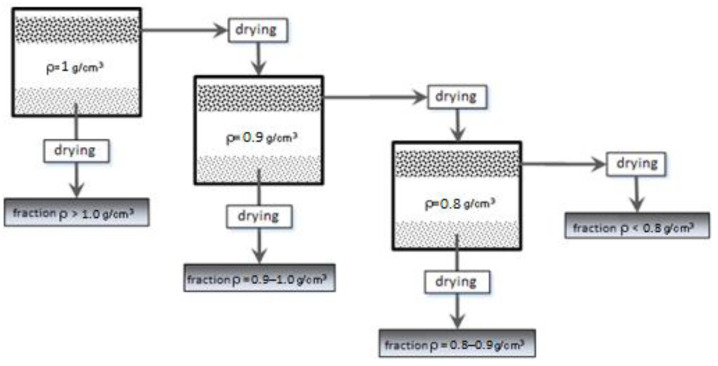
Scheme of the procedure for separating density fractions.

Dried microspheres were separated into density fractions in liquids with different densities. Portions of approximately 100 g were weighed, with an accuracy of 0.01 g, poured into an Imhoff sedimentation cone (Imhoff funnel) half-filled with water, and then filled with water to a volume of 1000 cm^3^. The suspension was thoroughly mixed and left to separate floating and sinking fractions. The fraction separation process took about 2 h, during which the suspension was thoroughly mixed every 30 min. After 2 h, both the material collected at the bottom of the funnel and the floating material were quantitatively transferred to stainless steel trays and dried in a thermal chamber at 80 °C until constant mass was achieved. After drying, the masses of the obtained fractions were weighed and recorded. Fractions with a density above 1 g/cm^3^ were further investigated, while the floating fraction was separated in a liquid with a density of 0.9 g/cm^3^. Further procedures followed the same steps as the separation of microspheres in water. After additional separations in liquids with densities of 0.9 and 0.8 g/cm^3^, three more fractions were obtained, namely with densities of 0.9–1.0 g/cm^3^, 0.8–0.9 g/cm^3^, and below 0.8 g/cm^3^. Liquids with densities lower than that of water were obtained by mixing ethanol or isopropanol with water in appropriate proportions. The scheme of density separation is presented in Figure 2.

(2) Separation of grain classes

The particle composition of the samples was made using selected sets of round laboratory sieves with square aperture (in µm): 500, 425, 315, 200, 160, 100, 90, 75, 56, and 40. Sieving of the samples was performed on a Multiserw (Mulitiserw-Morek, Brzeźnica, Poland) laboratory sieve shaker. Samples of microspheres, with a volume of approximately 100 cm^3^, were sieved for the same length of time and weighed with an accuracy of 0.01 g. The sieving time was 10 min, with a vibration amplitude of 20. After completing the sieving, the separated grain classes were weighed with an accuracy of 0.01 g. The content of the selected grain size classes was calculated as the ratio of the mass of microspheres retained on a sieve of a given size to the mass of the feed expressed as a mass percentage.

(3) True density

True density of the microspheres was determined using the helium method. Measurements were performed in an AccuPyc II 1340 pycnometer (Micromeritics, Norcross, GE, USA). The analyzer works based on the principle of gas buoyancy. It allows us to determine the density and actual volume by measuring changes in helium pressure between two chambers of a known and calibrated volume. The measurement takes into account the penetration of pores with a diameter greater than 10 nm. Analyses were conducted for a set equilibrium value of 0.005 psig and 20 measurement cycles.

(4) Analysis of the oxide composition using the XRF method

The samples of microsphere intended for testing were ground in a Fritsch Pulverisette 2 mortar grinder (FRITSCH GmbH, Weimar, Germany) until the fineness was below 75 µm. Before determining the oxide composition, the crushed samples of microsphere were subjected to the so-called “after ashing”, i.e., roasting at a temperature of 600 °C until a constant mass is obtained. The weight loss of the sample at this temperature expressed as a percentage was determined by the so-called Lost on Ignition—LOI600. Samples weighing 2 g were tested, which were ground with 0.5 g of wax in an agate crucible until a homogeneous mixture was obtained, and then the pellet was pressed under a pressure of 60 MPa. The chemical composition microspheres were determined with an ARL OPTIM’X WDXRF Spectrometer (Thermo Fisher Scientific, Waltham, MA, USA).

(5) Mechanical strength

The mechanical strength test usually consists of two elements: the selection of the material destruction method (compression, shear, or crushing) and the assessment parameters of the material before and after the destruction process—grain size distribution, sink–float test, etc. The methodology for determining the mechanical strength of microspheres adopted in our research is based on the method used by key recipients of microspheres.

The destructive factor in the strength testing method is the force exerted on the microsphere grains by the liquid in which they are immersed. The liquid pressure causes the same force, proportional to the liquid pressure, to act on all microsphere grains from all directions. The content of falling particles (referred to as sinkers) was assumed as the parameter defining the quality of microspheres before and after a destructive action, i.e., compression of microspheres using water pressure.

Determination of the content of falling particles (sinkers) consists of determining the mass fraction of the material collected in the bottom part of the Imhoff cone after 1 h from the moment of introducing 100 cm^3^ of microspheres into the cone, filling it up with water to the level of 1000 cm^3^, and thorough mixing of the content. To determine the mass fraction (content) of the sinkers, the sample is placed into the cone, and the sinkers are weighed with an accuracy of ±0.01 g (dry state). The content of falling particles in the sample in % provided by the supplier or in the sample before the strength test is called sinkers (S).

The analysis of mechanical strength of microspheres consists of exposing them to a water pressure of 3000 psi (20.7 MPa). This pressure is kept constant for 15 min, and the test is performed at ambient temperature. A 100 cm^3^ sample of microspheres weighed with an accuracy of ±0.01 g (dry state) is poured into a pressure vessel and filled with demineralized water to its entire volume. After closing and checking the tightness of the vessel, water is pumped using a high-pressure pump to reach a pressure of 3000 psi (approx. 20.7 MPa). This pressure is maintained in the vessel for 15 min. Then the pressure in the vessel is lowered until it equalizes with atmospheric pressure. The contents of the pressure vessel are then transferred to an Imhoff funnel, where the density separation of microspheres takes place using the “float-sink” method. Undamaged microspheres, being lighter than water, accumulate on its surface. The falling grains include particles damaged (crushed) as a result of the pressure during the test, as well as sinkers that existed in the sample from the beginning. The dried falling grains are weighed with an accuracy of 0.01 g. The mechanical strength *MS* is determined using the following formula:

$$MS=100\cdot \frac{m_{f}}{m_{s}}-S,\;\%$$

where:

*m_f_*—mass of falling grains after the mechanical strength test, g,
*m_s_*—mass of microspheres sample before mechanical strength test, g,
*S*—mass fraction of falling grains before mechanical strength test, %.

End users define the strength criteria for the microspheres they receive based on their own needs. For example, in the drilling industry, a product that meets the requirements of customers must have a strength lower than 30%. Microspheres with a strength of *MS* = 30–40% are accepted conditionally, while microspheres with a strength greater than 40% do not meet the requirements of the customer.

## 3. Results and Discussion

The results of the research are presented in three sections: basic properties of microspheres, densiometric and grain analysis, and strength for individual density fractions and grain classes.

### 3.1. Basic Properties of the Tested Microspheres

First, a series of routine tests were performed to characterize and evaluate the microsphere raw material. The basic properties of the tested microspheres included content of falling particles, true density, grain size distribution, and oxide composition. All tests in this section were performed for the microsphere raw materials—Material 1 and Material 2. 

Table 1, Table 2 and Table 3 present the results of the tests. The first set of data presented in Table 1 shows the results of falling particles content and true density determinations.

Material 1 exhibits a better performance than Material 2. The difference between the tested raw materials is more pronounced in terms of falling particles content, which is over twice as high for Material 2 compared to Material 1. The significantly higher density in the case of Material 2 is the result of a high content of falling particles.

Grain size distribution of the microspheres in the tested raw materials is presented in Table 2.

Analyzing the results of grain size distribution, it can be concluded that Material 1 2 is finer. The fraction with a size smaller than 75 µm has a mass fraction approximately 60% larger than in the case of Material 1. Material 2 does not contain particles larger than 315 mm, while the mass fraction of particles larger than 315 µm is small and does not exceed 2%. A clear difference is visible in the content of grain classes 200–315 µm and 160–200 µm. The total mass fraction of these two grain classes for Material 1 is 33.61%. In the case of Material 2, it is over 4 times lower. The common feature of both materials is the leading mass share of the fraction with a grain size in the range of 100–160 µm. Table 3 shows the results of the analysis of the oxide composition using the XRF method.

The mass fraction of individual oxides is at a similar level for the tested materials. The biggest differences can be seen in the content of two basic oxides that build microspheres, namely silicon dioxide and aluminum dioxide. Although in both cases, silicon dioxide is the oxide with the largest share and aluminum dioxide has the second largest share by mass, the proportions are different for each material. Material 1 is characterized by a higher share of SiO_2_ compared to Material 2 and, in turn, a lower mass fraction of Al_2_O_3_. The SiO_2_/Al_2_O_3_ proportion here is almost 2. The situation is different in the case of Material 2, for which this proportion is approximately 1.5. For comparison, studies on the chemical composition of microspheres were cited, including Haustein et al. [22], Strzałkowska et al. [1], and Feng et al. [33]. In the above studies, the ratio of these oxides is: 1.8 (for fractions < 200 µm), 1.40–1.72 (from different types of coal), and 1.27–1.39 (from different types of carbon), respectively. The chemical composition of the oxides in the tested samples does not differ from the data published in the literature. Generally, the chemical composition of the microspheres always maintains the condition of SiO_2_ > Al_2_O_3_, while the content of the remaining oxides does not show such proportions. It should be emphasized, however, that the ratios of the individual oxides determine the properties of the microspheres. According to the work of [34], the color of the microspheres depends on the ratio of the content of SiO_2_ and Fe_2_O_3_ to Al_2_O_3_. Cenospheres, which are rich in Al_2_O_3_ and have a low content of SiO_2_ and Fe_2_O_3_, are white. Those with a high percentage of SiO_2_ and Fe_2_O_3_ become gray.

### 3.2. Densiometric Analysis

Density is one of the quality parameters of microspheres. The specific density of microspheres was tested both in the raw material and in individual grain classes. 

Table 4 summarizes the results of the densiometric analysis for Material 1.

Analyzing the obtained results, an interesting phenomenon should be noted: in the grain class < 75 μm, grains with relatively high density accumulate in comparison to larger grain classes. This is particularly visible in the case of fractions with higher densities, i.e., starting from the fraction 0.8–0.9 g/cm^3^. For these cases, the density of the finest grain class is higher than all the others. For the raw material, the situation is similar, with the difference, however, that the density of the finest fraction is higher than that of almost all grain classes except those in the range of 200–315 μm. In fact, only in the case of fractions with a density lower than 0.8 g/cm^3^ can the following relationship be observed: as the grain size decreases, the specific density of a given grain class decreases. Based on these results, it should be assumed that a significant part of the finest grain class has a feature or features that cause its high density. Observations made during the research indicate that this may be the result of differences in the structure of the walls of microspheres in different grain classes in terms of their thickness or chemical composition. However, a full assessment of this issue requires separate research.

Generally speaking, it should be stated that the dominant grain class for the raw material and individual density fractions is in the range of 100–160 μm. The exception to this rule is the density fraction greater than 1 g/cm^3^, in which the mass fraction of the finest grain class is approximately 67%. This grain class is also characterized by a relatively high density. As for density fractions, the model fraction seems to be the fraction with a density below 0.8 g/cm^3^. The remaining ones tend to accumulate relatively high-density particles in the smallest grain class. 

**Figure 3:**
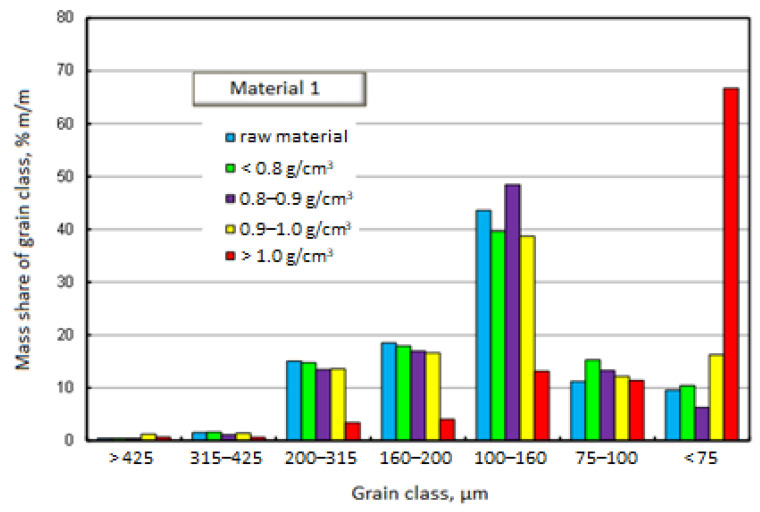
Mass fractions of separated grain size classes with specification of density fractions—Material 1.

**Figure 4:**
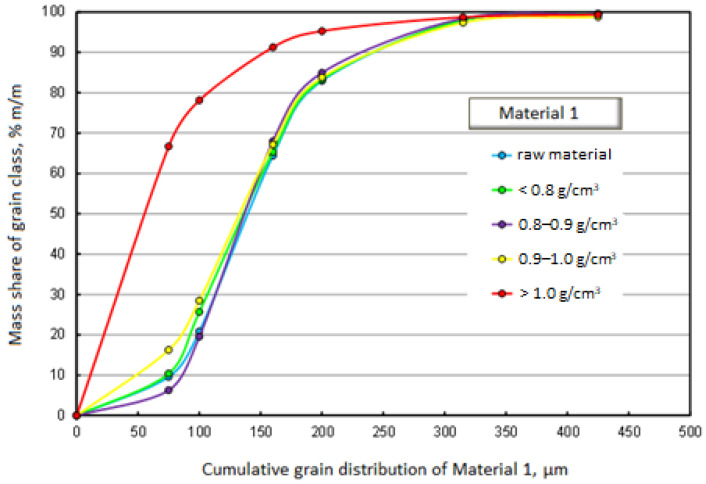
Cumulative grain size distribution of the raw material and separated density fractions—Material 1.

Figure 3 and Figure 4 graphically present the results of the densiometric and grain analyses. In the first case, it is a chart showing the mass shares of separated grain classes with specification of density fractions. The second drawing shows the cumulative grain distribution of the raw material and the separated density fractions.

Figure 3 and Figure 4 show that the grain distributions of the separated density fractions and of the raw material itself are similar. The exception is the fraction falling in water, i.e., the fraction with a density above 1 g/cm^3^. As much as 70% by mass of this fraction belongs to the grain class below 75 μm. Although the mass share of the fraction with a density above 1 g/cm^3^ is not large (3% m/m), accumulating a significant majority of its mass in the smallest grain class is a very serious problem. The share of this density fraction increases the specific density of the products, reducing their quality. 

The raw material of Material 1 is a relatively fine material. This is evidenced by, among others, 90% mass fraction of grains with a size below 250 μm, and 50% mass fraction of grains below 150 μm. The dominant grain class in all separated density fractions, except for the fraction with a density above 1 g/cm^3^, is the class of 100–160 μm. 

Table 5 summarizes the results of the densiometric analysis for Material 2.

The dominant grain class, as in the case of Material 1, is in the range of 100–160 μm. The difference between the two materials in this context is that there is no exception for fractions with a density greater than 1 g/cm^3^. Comparing the analysis results for both materials, it can be noticed that Material 2 is finer than Material 1. This applies both to the raw material, which has already been indicated in the grain size analysis, and to individual density fractions. The mass fraction of grain classes with a size larger than 160 μm is definitely lower than in the case of Material 1. Fractions with a density above 0.8 g/cm^3^ tend to accumulate particles of relatively high density in the smallest grain classes. 

The results of the analysis indicate another puzzling feature of these microspheres, which, with one exception, was not revealed in the case of Material 1. Namely, the specific density in individual grain classes in relation to the density fraction. In most cases, the density in the grain classes is higher than the density fraction to which they were assigned on the basis of the division. This situation occurs for each grain class within three density fractions, i.e., 0.8–0.9 g/cm^3^, 0.9–1.0 g/cm^3^, and above 1.0 g/cm^3^, as well as for one grain class classified in the density fraction lower than 0.8 g/cm^3^. This is a different situation than in the case of Material 1, for which this type of exceedance occurred only for the grain class < 75 μm within the density fraction of 0.8–0.9 g/cm^3^. This phenomenon may have various causes. The reason may be the property indicated above for the high specific density noted for the smallest grain class, namely the differentiation of the wall structure. The frequency of this phenomenon is significant, suggesting the existence of other factors influencing such results.

In the case of Material 2, the same tendency is observed as for Material 1, namely the accumulation of grains with a relatively high density in the grain class < 75 μm compared to larger grain classes. Similarly, it is noticeable from the fraction of 0.8–0.9 g/cm^3^ upwards. In these cases, the density of the finest grain class is higher than all the others. For the raw material, the situation is similar, with the difference that the density of the finest fraction is higher than that of almost all grain classes except those in the range of 160–200 μm. For fractions with a density lower than 0.8 g/cm^3^, this relationship is almost the same as for Material 1, with a slight increase in density for the smallest grain classes compared to slightly larger grains with sizes in the range of 75–100 μm, distinguishing nothing in this regard for both materials. However, a full assessment of this issue requires a number of laboratory tests. 

**Figure 5:**
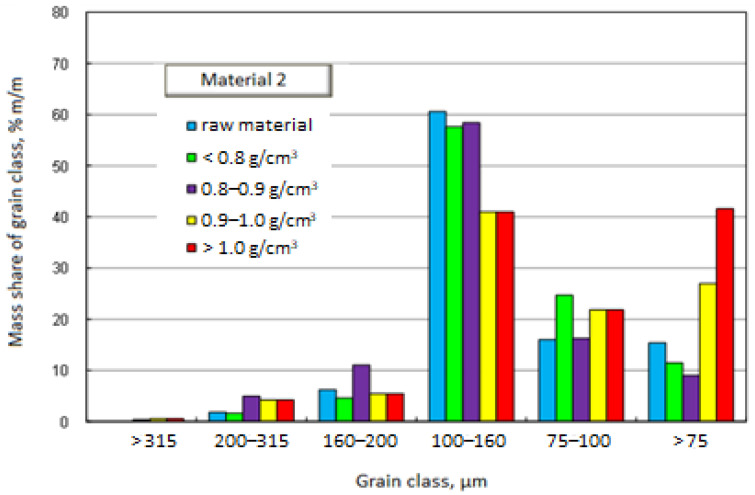
Mass fractions of separated grain size classes with specification of density fractions—Material 2.

**Figure 6:**
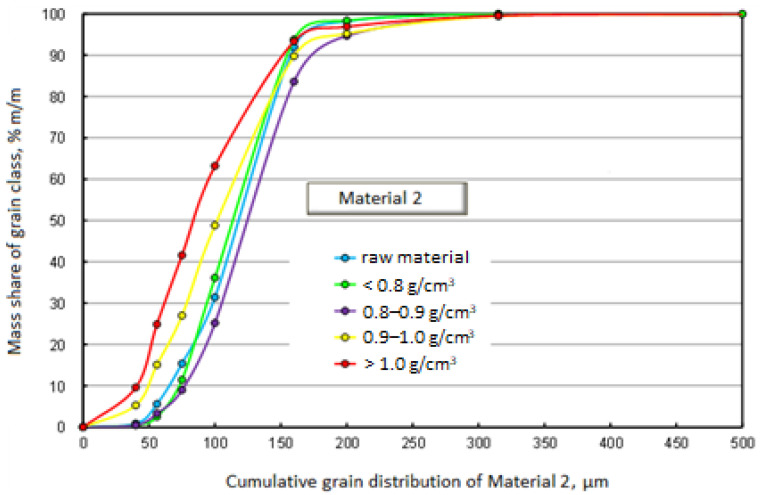
Cumulative grain size distribution of the raw material and separated density fractions—Material 2.

Figure 5 and Figure 6, similarly to Material 1, graphically present the results of densiometric and grain analysis.

In the case of Material 2, similarly to Material 1, the grain distributions of the density fractions and the raw material itself are similar. Unlike Material 1, the grain distribution of the fraction with a density above 1 g/cm^3^ does not differ significantly from the remaining density fractions and the raw material. Such a grain composition of fractions with a density above 1 g/cm^3^ will result in an even load on all grain classes, without causing accumulation in one of the classes. The dominant grain class in all separated density fractions, as in the case of Material 1, is 100–160 μm.

### 3.3. Investigation on the Mechanical Strength of Microspheres in Density Fractions

The results of the mechanical strength test of the microsphere raw Material 1 and its fractions with a density less than 0.8 g/cm^3^ are presented in Table 6. Grain size classes with good (*MS* < 30%) and acceptable (*MS* = 30–40%) for drilling industry mechanical strength are highlighted here in green.

The test results indicate an increasing trend in the strength of microspheres with decreasing grain class size. The upward trend does not cover the smallest grain classes tested, i.e., grains with dimensions below 75 μm. This correlation is similar to that previously observed in densiometric analysis, although it did not include fractions with a density below 0.8 g/cm^3^. In the case of raw Material 1, the finest grain class has significantly lower mechanical strength than the same grain class separated from this raw material with a density fraction below 0.8 g/cm^3^. This fact refers to the previously carried out densiometric and grain analyses of raw Material 1. They showed that the initial content of the falling particles with a density above 1 g/cm^3^ in the raw material is 3% m/m. Of this amount, over 60% have dimensions below 75 μm. Therefore, the grain class of raw Material 1 with dimensions below 75 μm does not have lower strength but is already loaded with ballast in the form of the initial content of falling particles. When separating fractions with a density below 0.8 g/cm^3^ from the raw material, the entire ballast is in the form of particles with a density above 1.0 g/cm^3^, but also particles with a density of 0.8–1.0 g/cm^3^. Hence, the strength of the microspheres in the crushing test of the grain class below 75 μm of the raw Material 1 is only apparently lower. The actual strength of the microspheres is reflected in the result obtained for a grain class of less than 75 μm sifted from material with a density of less than 0.8 g/cm^3^. We observe similar relationships for grain classes below 160 μm.

Undamaged microspheres with dimensions below 160 μm exhibit very high and very desirable mechanical strength. The problem of obtaining such durable microspheres results from the fact that during refining processes, the microspheres are partially degraded and, together with the initial ballast, i.e., the falling particles, they move to finer grain classes of products, relatively reducing their strength indicators. 

The opposite tendency regarding strength indicators can be observed in the case of microspheres with dimensions above 160 μm. Strength indicators for coarse grain classes of the raw material are slightly better (by 5 percentage points) than for the corresponding grain classes with a density below 0.8 g/cm^3^. In this case, the explanation should most likely be sought in the structure of the microsphere grains and the mechanism of strength testing. 

Table 7 shows the strength test results for Material 2.

Analyzing the results of the strength tests, it can be inferred that raw Material 2 exhibits weak strength parameters. In the raw material, only the grain class of 75–100 µm constitutes acceptable material. The separated density fraction with a density below 0.8 g/cm^3^ does not significantly differ in terms of mechanical strength from the raw material from which it was separated. Only the grain classes of 75–100 µm and below 75 µm represent acceptable material.

The fraction with a density below 0.8 g/cm^3^ does not contain ballast in the form of primary falling particles contained in the raw material. It can be assumed, therefore, that the strength testing of microspheres with a density below 0.8 g/cm^3^ directly reflects their mechanical strength. Hydraulic compression of microspheres for 15 min under a pressure of 3000 psi causes some of them to undergo mechanical damage. Part of the microspheres, possessing pores accessible to water under high pressure during the test, increase their density and sink in water. It should be assumed that the effect of pore filling by water is the dominant factor affecting the strength indicators for microspheres with dimensions above 100 µm.

## 4. Conclusions

Preparing a final product from microsphere raw material with the properties expected by the end user requires an appropriately selected refining process. The key to the optimal selection of the sequence and parameters for a grain separation operation is the knowledge of the characteristics of the processed raw material. Microspheres are materials that are created spontaneously, uncontrolled, and without the possibility of intentionally controlling their properties during their formation. Therefore, due to the potential directions of use of microspheres, it is justified to study the relationship between specific density, grain size, and mechanical strength. 

The results of testing microsphere raw materials from two different sources are presented. A comparative analysis of the basic properties of the tested microsphere raw materials showed that more favorable operational parameters, from a commercial point of view, have Material 1, containing 3% falling particles (*S*) and low specific density (0.7133 g/cm^3^). 

The tested microsphere raw materials were subjected to densiometric and grain analysis. Mechanical strength and specific density were determined for grain classes separated from density fractions. Practically, for both tested materials and all density fractions separated from them, it was shown that the dominant grain class in terms of mass share is the class with a grain size of 100–160 μm. In turn, the grain class characterized by the highest mechanical strength for both materials is the 75–100 μm class. Within this grain size range, the mechanical strength is 26 for raw Material 1 and 38 for raw Material 2. The proportions of this grain fraction in the microsphere stream are 11.2% and 16%, respectively. Comparing the grain distributions and densities of grain classes, a very unfavorable tendency can be noticed, consisting of the accumulation of small particles with high specific density in the smallest grain classes. This regularity causes particles with the highest specific density to accumulate in the grain class containing microspheres with the highest mechanical strength, reducing the resultant strength. The reduction in strength is only apparent and results from the adopted methodology for assessing the strength of microspheres. The conducted research confirmed certain regularities regarding the characteristic properties of microspheres but did not reveal any significant correlations between the tested features of the microspheres. The test results showing the distribution of primary falling particles (sinkers—*S*) between density fractions and grain classes constitute valuable information helpful in selecting appropriate operating parameters for installations refining microsphere raw materials.

## Figures and Tables

**Table 1:** Basic properties of the microspheres.

Parameter	Unit	Material 1	Material 2
Content of falling particles (sinkers) in raw material	% m/m	3	7
True density in raw material	g/cm^3^	0.7133	0.8275

**Table 2:** Grain distribution of the tested microspheres.

Material 1		Material 2	
Size of the Grain Class, µm	Share of the Grain Class, % m/m	Size of the Grain Class, µm	Share of the Grain Class, % m/m
>425	0.46	-	-
315–425	1.51	315–425	0.00
200–315	15.07	200–315	1.80
160–200	18.54	160–200	6.20
100–160	43.58	100–160	60.60
75–100	11.24	75–100	16.00
<75	9.60	56–75	9.8
-	-	40–56	4.70
-	-	<40	0.90

**Table 3:** Oxide composition of the tested microspheres.

Parameter		Unit	Material 1	Material 2
Silicon dioxide	SiO_2_	% m/m	59.07	55.20
Aluminum dioxide	Al_2_O_3_	% m/m	29.54	37.10
Diiron trioxide	Fe_2_O_3_	% m/m	1.84	2.30
Calcium oxide	CaO	% m/m	1.98	1.52
Magnesium oxide	MgO	% m/m	0.38	0.51
Diphosphorus pentoxide	P_2_O_5_	% m/m	0.71	0.86
Sulfur trioxide	SO_3_	% m/m	0.13	0.09
Trimanganese tetraoxide	Mn_3_O_4_	% m/m	0.04	0.06
Titanium dioxide	TiO_2_	% m/m	1.07	0.98
Barium oxide	BaO	% m/m	0.42	0.21
Strontium oxide	SrO	% m/m	0.11	0.08
Disodium oxide	Na_2_O	% m/m	0.56	0.38
Dipotassium oxide	K_2_O	% m/m	0.60	0.51

**Table 4:** Share of grain classes and their densities in density fractions—Material 1.

Grain Class	Raw Material		<0.8 g/cm^3^		0.8–0.9 g/cm^3^		0.9–1.0 g/cm^3^		>1.0 g/cm^3^	
	Share %	Density g/cm^3^	Share %	Density g/cm^3^	Share %	Density g/cm^3^	Share %	Density g/cm^3^	Share %	Density g/cm^3^
>425	0.5	n/o	0.4	n/o	0.5	n/o	1.2	n/o	0.7	n/o
315–425	1.5	n/o	1.6	0.7901	1.1	n/o	1.4	n/o	0.6	n/o
200–315	15.1	0.7415	14.8	0.7048	13.5	0.8989	13.6	0.9608	3.5	1.3943
160–200	18.5	0.7186	17.9	0.6559	16.9	0.8852	16.6	0.9585	4.0	1.4999
100–160	43.6	0.7127	39.6	0.6531	48.5	0.8789	38.7	0.9301	13.2	1.4121
75–100	11.2	0.7020	15.3	0.6488	13.3	0.8799	12.2	0.9293	11.4	1.6271
<75	9.6	0.7305	10.4	0.6278	6.3	0.9347	16.3	0.9863	66.7	1.8580

**Table 5:** Share of grain classes and their densities in density fractions—Material 2.

Grain Class	Raw Material		<0.8 g/cm^3^		0.8–0.9 g/cm^3^		0.9–1.0 g/cm^3^		>1.0 g/cm^3^	
	Share %	Density g/cm^3^	Share %	Density g/cm^3^	Share %	Density g/cm^3^	Share %	Density g/cm^3^	Share %	Density g/cm^3^
>315	0.0	n/o	0.0	n/o	0.4	n/o	0.5	n/o	0.5	n/o
200–315	1.8	n/o	1.6	1.0009	4.9	1.0009	4.2	1.1505	2.6	n/o
160–200	6.2	0.7415	4.6	0.7889	11.1	0.9061	5.4	1.1622	3.5	1.5482
100–160	60.6	0.7186	57.6	0.7477	58.4	0.9724	41.0	1.3327	30.2	1.8644
75–100	16.0	0.7127	24.7	0.6987	16.2	1.0023	21.8	1.5177	21.6	1.8648
56–75	9.8	0.7020	8.9	0.7099	5.7	1.0325	11.9	1.6790	16.7	1.9754
40–56	4.7	0.7305	2.0	0.7099	2.8	1.0475	9.8	1.8997	15.3	2.1048
<40	0.9	0.7305	0.2	0.7099	0.5	n/o	5.3	2.1861	9.6	2.2487

**Table 6:** Mechanical strength (*MS*) of raw material and fractions with density < 0.8 g/cm^3^ in grain classes—Material 1. Good and acceptable mechanical strength values are highlighted in green.

Grain Class	Raw Material		<0.8 g/cm^3^	
	Share %	Strength %	Share %	Strength %
315–425	1.5	70	1.6	-
200–315	15.1	52	14.8	57
160–200	18.5	36	17.9	41
100–160	43.6	27	36.9	30
75–100	11.2	26	15.3	24
<75	9.6	37	10.4	26

**Table 7:** Mechanical strength (*MS*) of raw material and fractions with density < 0.8 g/cm^3^ in grain classes—Material 2. Good and acceptable mechanical strength values are highlighted in green.

Grain Class	Raw Material		<0.8 g/cm^3^	
	Share %	Strength %	Share %	Strength %
>160	8.0	59	6.2	61
100–160	60.6	42	57.6	42
75–100	16.0	38	24.7	30
<75	15.4	48	11.5	34

## Data Availability

The original contributions presented in the study are included in the article.
